# Do Uncontrolled Hypertension, Diabetes, Dyslipidemia, and Obesity Mediate the Relationship Between Health Literacy and Chronic Kidney Disease Complications?

**DOI:** 10.3390/ijerph18105235

**Published:** 2021-05-14

**Authors:** Matheus Gurgel do Amaral, Sijmen A. Reijneveld, Josue Almansa, Gerjan Navis, Andrea F. de Winter

**Affiliations:** 1 Department of Health Sciences, Community and Occupational Medicine, University Medical Center Groningen, Hanzeplein 1, 9700RB Groningen, The Netherlands; s.a.reijneveld@umcg.nl (S.A.R.); j.almansa.ortiz@umcg.nl (J.A.); a.f.de.winter@umcg.nl (A.F.d.W.); 2 Department of Nephrology, University Medical Center Groningen, Hanzeplein 1, 9700RB Groningen, The Netherlands; g.j.navis@umcg.nl

**Keywords:** health literacy, chronic kidney insufficiency, cardiovascular disease, mortality, diabetes mellitus, obesity

## Abstract

Health literacy is the ability to deal with information related to one’s health. Patients with low health literacy and chronic diseases, such as chronic kidney disease (CKD), have poor disease-management skills, which could lead to complications. We used logistic regressions and structural equational modeling to assess whether low health literacy is associated with the development of cardiovascular disease and mortality in patients with CKD, and whether this association is mediated by the presence of uncontrolled hypertension, diabetes, dyslipidemia, obesity, or albuminuria. Data from 2742 adult participants with CKD from the Lifelines study were analyzed at baseline and after approximately four years. Low health literacy was associated with cardiovascular disease and mortality in the crude models, with OR and 95%CI of 1.93 (1.46 to 2.55) and 1.59 (1.08 to 2.36), respectively. After adjustment for age and sex, low health literacy was only associated with cardiovascular disease (OR 1.76 (1.31 to 2.23)). This association was mediated by uncontrolled diabetes (27.1%) and obesity (8.0%). Low health literacy is associated with the development of cardiovascular disease after adjustment for age and sex, and this association is mediated by uncontrolled diabetes and obesity.

## 1. Introduction

Health literacy is one of the main social determinants of health because of its negative effects on many chronic diseases [1], such as chronic kidney disease (CKD). Health literacy can influence health behaviors and disease management [2], affecting the progression of CKD [3]. Therefore, enhancing our understanding of the association between health literacy and CKD is important for promoting better CKD care.

Health literacy is defined as the degree to which individuals have the capacity to obtain, process, and understand basic health information needed to make appropriate health decisions [4]. Low health literacy is a highly prevalent problem worldwide [5] and has been associated with a variety of negative outcomes, including a suboptimal utilization of health services [6], increased mortality [1], and higher healthcare costs [7]. Some patients with low health literacy experience difficulties even in simple commonplace activities, such as reading a leaflet, keeping track of medical appointments, or identifying how often a person should have a specific medical test [8]. Thus, individuals with low health literacy lack the ability to properly manage their diseases and prevent complications.

Complications associated with CKD, such as cardiovascular disease and mortality, are more prominent among individuals with low health literacy [3,9]. The pathways underlying the relationship between low health literacy and CKD complications, however, are still unclear. A key to understanding these pathways may lie in the control of some of the CKD treatment targets, such as hypertension, diabetes, dyslipidemia, obesity, and albuminuria [10]. These treatment targets, often mentioned in CKD management guidelines [10], are less optimally managed by people with low health literacy and could mediate the relationship with CKD complications [11,12,13].

A clearer understanding of how low health literacy could hinder the management of CKD treatment targets, which could lead to the development of CKD complications, is lacking because the literature on the topic regards mostly cross-sectional studies or cohorts assessing only dialysis patients. Prospective studies also including earlier stages of CKD, where preventive efforts are more efficient [10], are still needed.

This study aims to assess whether low health literacy is associated with the development of cardiovascular disease and mortality in patients with CKD, and whether this association is mediated by the presence of uncontrolled hypertension, diabetes, dyslipidemia, obesity, or albuminuria. Understanding the role of management of these CKD treatment targets could be a first step toward the development of measures to reduce the impact of low health literacy on CKD complications.

## 2. Materials and Methods

### 2.1. Sample and Procedures

The study sample was derived from the first (T1) and second (T2) assessment waves of the Lifelines study. Lifelines is a multi-disciplinary, prospective, population-based cohort study examining, in a unique three-generation design, the health and health-related behaviors of 167,729 people living in the north of the Netherlands. It employs a broad range of investigative procedures in assessing the biomedical, socio-demographic, behavioral, physical, and psychological factors which contribute to the health and disease of the general population, with a special focus on multimorbidity and complex genetics. Data collection for adults was performed between November 2006 and December 2013 for T1 (*n* = 152,737) and between January 2014 and July 2018 for T2 (*n* = 111,915). The recruitment and collection of the data has been described in detail elsewhere [14]. The Lifelines Cohort Study was conducted in accordance with the principles of the Declaration of Helsinki and the research code of the University Medical Center Groningen [14]. Exclusion criteria for our study included pregnancy and a history of kidney transplantation, due to the changes in renal function and prognosis that these conditions entail [15,16].

Our longitudinal study used information from T1 and T2. Participants were eligible for our study if they were 18 or older, had completed the health literacy questionnaire, and could be classified as having CKD based on their albuminuria and serum creatinine levels.

### 2.2. Measurements

#### 2.2.1. Health Literacy

Health literacy was measured at T1, using self-reported answers to the three validated questions from Chew et al. [17]:

1. How often do you have trouble understanding your medical situation because you have difficulty with the written information?
2. How sure are you of yourself when you fill out medical forms?
3. How often does someone help you with reading information materials from the hospital or another healthcare provider?

Participants answered these questions on a Likert scale ranging from 1 (Never/not at all) to 5 (Always/Very). We reversed the scores of the first and third questions and then added up the scores of all questions, resulting in a health literacy scale ranging from 3 to 15. Participants who scored 3 to 12 points were considered to have low health literacy and participants with 13 or more points were considered to have adequate health literacy. This cut-off point is in accordance with that used in previous studies using the same database [18] and leads to percentages of low and adequate health literacy comparable to those found in large-scale health literacy surveys in the Netherlands [19].

#### 2.2.2. Definition of Chronic Kidney Disease

In accordance with the latest guidelines [10], a participant needed to meet at least one of the following criteria at T1 in order to be classified as having CKD:

4. Estimated glomerular filtration rate (eGFR) lower than 60 mL/min/1.73 m^2^;
5. Albuminuria assessed by 24 h urine ≥ 30 mg/24 h or assessed by albumin-to-creatinine ratio in morning urine ≥ 3 mg/mmol.

The eGFR was calculated with the CKD-EPI formula [10,20], which uses values of serum creatinine, age, and sex. Creatinine levels were assessed with serum laboratory tests performed on fasting blood samples drawn from participants at one of the Lifelines research sites. On the same day, participants had to hand in urine samples collected at home. The assessment of albuminuria by Lifelines stopped midway at T1 due to logistic reasons. Therefore, in the sample of adults who completed the health literacy questionnaire, 54% did not have values of albuminuria, and only their eGFR was used in the definition of CKD.

#### 2.2.3. Cardiovascular Disease and Mortality

Cardiovascular disease (y/n) is a composite outcome consisting of the self-reported history (y/n) of new myocardial infarction, heart failure and/or stroke. It was assessed three times during the T2 wave. People who had no cardiovascular disease at T1 but reported a new diagnosis at any point of T2 were considered as positive for this outcome. ECGs were not used to define new myocardial infarction or new heart failure because abnormalities in the ECG are not sufficiently specific without the patient’s clinical history and a medical diagnosis. Mortality (y/n) was defined as all-cause mortality at any time from the baseline assessment until November 2018 and was retrieved from the Dutch CBS registry [21].

#### 2.2.4. CKD Treatment Targets

The CKD treatment targets studied as mediators were uncontrolled hypertension, diabetes, dyslipidemia, obesity, and albuminuria. Being uncontrolled meant that their values were above the recommended limit in the treatment of CKD patients. Uncontrolled hypertension was defined as having blood pressure out of the target according to guidelines specific for the participants’ age, diabetes and albuminuria status [10]. Uncontrolled diabetes was defined as having glycated hemoglobin (HbA1c) above 6.5%. We used single measurements of blood pressure and HbA1c to assess uncontrolled hypertension and diabetes because, according to the central limit theorem, individual fluctuations in blood pressure and HbA1c would not have a big influence on the final estimate in such a large sample size. Dyslipidemia was assessed with the cholesterol ratio, which is calculated by dividing the total cholesterol levels by the high-density lipoprotein (HDL) cholesterol levels and considered uncontrolled if above 5 in men and 4.5 in women. For the study of obesity, BMI was dichotomized and considered high if above 30 kg/m^2^. Uncontrolled albuminuria was defined as albuminuria assessed by 24 h urine ≥ 30 mg/24 h or assessed by albumin-to-creatinine ratio in morning urine ≥ 3 mg/mmol. Glycated hemoglobin, total cholesterol, HDL, and low-density lipoprotein (LDL) levels were obtained with serum laboratory tests performed on fasting blood samples drawn from participants at one of the Lifelines research sites. On the same day, participants had their blood pressure, height, and weight measured by trained technicians. All variables were assessed at T1.

#### 2.2.5. Other Variables

Sex, age, educational level, monthly household income, and current smoking were reported by the participants in a questionnaire applied at T1. Age was categorized as younger than 45, between 45 and 65, and over 65 years. Educational level was measured with an 8-item ordinal scale (from *No education* to *University education*). The answers to this questionnaire were posteriorly categorized as low (consisting of no education or complete primary education), intermediate (consisting of complete secondary education), and high (consisting of higher vocational education or university education). Monthly household income was measured with an 8-item ordinal scale (from less than 750 euros to more than 3500 euros), and I do not know or I would rather not answer this question. The answers were clustered into four categories: less than 1000 euros, 1000–3000 euros, more than 3000 euros, and information not given. Participants were defined as current smokers if they reported any smoking in the previous month. We also wanted to include smoking as a potential mediator, but this variable was excluded due to methodological limitations.

### 2.3. Analysis

Firstly, we calculated descriptive statistics and evaluated differences between the low- and adequate-health-literacy groups using Pearson’s chi-square tests, independent sample *t*-tests, or Mann–Whitney tests. Secondly, we assessed if low health literacy was associated with cardiovascular disease and mortality using logistic regression. Thirdly, we assessed whether uncontrolled hypertension, diabetes, dyslipidemia, obesity, or albuminuria mediated the relationships above. We solved this by using probit structural equation modeling to explore the direct and indirect, i.e., mediated, association between health literacy and cardiovascular disease and mortality. The strength of the mediation effect was expressed in terms of percentage of the association explained by the mediator. All the models above were adjusted for age and sex. The analyses were conducted with IBM SPSS Statistics version 22 and R version 3.4.2 (package lavaan) for Windows and the results of all models were considered statistically significant if *p* < 0.05.

#### Sensitivity Analysis

We performed five sensitivity analyses. First, we repeated the logistic regressions including eGFR as an extra confounder. This was performed because people with low health literacy present worse eGFR levels [3], which could explain the association with CKD complications. Second, we tested the association between low health literacy and mortality using Cox proportional hazards modeling. This type of analysis was not possible for the association with cardiovascular disease because this outcome was measured only three times. Third, we repeated the mediation analyses using the mediators as continuous variables, namely systolic blood pressure, diastolic blood pressure, serum HbA1c, BMI, cholesterol ratio, and 24 h albuminuria. Fourth, we repeated the mediation analysis, including all the mediators simultaneously in the same model, to check if correlations between them would affect the results. Fifth, we repeated the mediation analyses for uncontrolled hypertension, diabetes, and dyslipidemia, also including treatment status as an extra confounder. Treatment status (y/n) referred to whether the individual was receiving treatment for the specific mediator tested; it was assessed at T1 with a questionnaire about medication use. Participants also had to bring in a detailed list of current medication. The types of medication by disease that we used in our sensitivity analysis can be found in the Supplementary Material.

## 3. Results

### 3.1. Population Characteristics

Sixty-five percent of the adult Lifeline participants answered the health literacy questionnaire. These individuals had lower rates of cardiovascular disease (11.9% versus 37.0%) than the ones who did not answer the questionnaire. They did not differ regarding mediators or confounders. The prevalence of CKD was 2.8% in the total Lifelines sample and 2.9% among individuals who answered the health literacy questionnaire (1.2% due to low eGFR and 1.8% due to albuminuria, i.e., 0.1% sufficed both criteria for CKD). The resulting sample comprised 2742 participants with a mean follow-up time of 4.2 years (standard deviation: 1.2 years). Data on cardiovascular disease and proteinuria were not available for 28% and 23% of the eligible sample, respectively. These were the only variables with more than 5% of missing values, and the groups with and without missing values did not show important differences. Ninety-nine percent of the participants had initial renal function compatible with milder renal impairment (36.3% in stage 1, 22.9% in stage 2, and 39.8% in stage 3) and 33.8% of them were classified as having low health literacy. Participants with low health literacy were more likely to be older, to have a worse renal function, and lower educational level and income. These and other clinical characteristics are presented in Table 1.

### 3.2. The Association between Low Health Literacy and CKD Complications

Low health literacy was associated with cardiovascular disease and mortality in the crude models (Table 2). Adjusted for age and sex, low health literacy was still associated with cardiovascular disease, but not with mortality.

### 3.3. The Mediation of Uncontrolled Hypertension, Diabetes, Obesity, Dyslipidemia, and Albuminuria

In the crude models, the association between low health literacy and cardiovascular disease was mediated by uncontrolled diabetes and obesity. The association between low health literacy and mortality was mediated by uncontrolled diabetes. After adjustment for confounders, only diabetes mediated the association between low health literacy and cardiovascular disease, and obesity showed the same amount of mediation but with a *p*-vale just above the level of significance. (Table 3)

### 3.4. Sensitivity Analysis

When the mediators were analyzed as continuous variables, the crude associations of health literacy with cardiovascular disease and with mortality were mediated by systolic blood pressure, HbA1c, BMI, and albuminuria 24 h. After adjustment for age and sex, the association between health literacy and cardiovascular disease was only mediated by BMI (9.4% of the association explained by BMI, *p* = 0.004), and there was no mediation between low health literacy and mortality. All other sensitivity analyses did not change the results.

## 4. Discussion

### 4.1. Main Findings

This study showed that, among patients with CKD, low health literacy was associated with cardiovascular disease and mortality in the crude analysis, but only with cardiovascular disease after adjustment for age and sex. The association with cardiovascular disease was clearly mediated by uncontrolled diabetes (27%) and, to a lesser extent, by obesity (8%).

We found that low health literacy was associated with a new diagnosis of cardiovascular disease over a period of approximately four years. This finding is in line with other studies performed in the CKD population [3], and could be due to the suboptimal disease self-management skills and utilization of health services present in individuals with low health literacy [6,22]. Our results, however, have special importance given the relatively healthy profile of the Lifelines population. Ninety-nine percent of our sample were people in CKD stages 1, 2, or 3, i.e., concerned mild kidney impairment, which implies a low risk of complications [23]. The association between low health literacy and the development of cardiovascular disease, even in the early stages of CKD, shows that measures to support patients with low health literacy may be essential to prevent CKD complications.

Low health literacy was associated with all-cause mortality in the crude analysis, but not after adjusting for age and sex; the latter contrasts with other studies performed with CKD patients. This contrast could be due to important differences in those studies when compared to ours [9,24]. While they focused on end-stage renal disease, our sample concerned individuals with milder CKD. Associated with that, our sample had a lower mortality rate, a lower rate of comorbidities, and a lower average age. Our findings suggest, therefore, that the association between low health literacy and mortality is limited to the most severe stages of CKD. This might happen either because the negative effects of low health literacy are not strong enough to cause death in relatively healthy patients, or because low health literacy has a cumulative effect. Thus, a longer follow-up would be needed to detect its impacts on mortality.

Uncontrolled diabetes partially mediated the association between low health literacy and cardiovascular disease in the adjusted analysis, and obesity was very close to significance. In the sensitivity analysis, when the mediators were analyzed as continuous variables, the mediation by BMI was significant, albeit small. Uncontrolled diabetes and obesity were identified as possible mediators because, compared to the other treatment targets tested, they are more responsive to lifestyle changes. The control of dyslipidemia and albuminuria, for instance, is more responsive to the pharmacological treatment than it is to lifestyle changes [25,26]. Conversely, a poor glycemic control is usually associated with insulin resistance and, therefore, more responsive to lifestyle changes than to changes in medication [27]. The control of blood pressure, in turn, can respond to both strategies to a certain extent [28]. Since low health literacy is associated with difficulties keeping up with healthy lifestyles and adhering to treatment regimens, it is logical that the association with cardiovascular disease would be mediated by the treatment targets more responsive to lifestyle changes. None of the treatment targets, however, worked as mediators in the association between low health literacy and mortality. This was probably due to the very low mortality rate of our sample, which did not yield enough power to assess mediation.

### 4.2. Strengths and Limitations

An important strength of this study was its large sample size with a longitudinal design. Furthermore, the population-based sample, which largely consisted of middle-aged participants and participants with milder renal impairment, is likely more comparable to the general populations. We also used an objective measurement of renal function and retrieved mortality data from an up-to-date registry, which probably led to a reduction in disease misclassification and reporting bias. Finally, we performed various sensitivity analyses that led to similar results, which endorsed the robustness of our findings.

This study also had limitations. First, we measured health literacy and cardiovascular disease based on self-report, which could have led to misclassification and, thus, to a larger measurement error and some underestimation of associations. Furthermore, given the intricate management of CKD, it could be that the more complex aspects of health literacy not measured in the questionnaire (namely, communicative and critical health literacy [29]) might also play an important role in the development of CKD complications. Nonetheless, our instrument is a validated tool [17,30] that has been used in other studies [31,32,33]. Third, 54% of our sample did not have values of albuminuria, which might have undermined the strength of the associations analyzed. Nevertheless, no important selection bias is expected because the values of albuminuria were obtained from a random subgroup of the Lifelines sample and its assessment stopped due to logistical reasons not related to CKD. Fourth, 65% of the participants answered the health literacy questionnaire. This probably reduced the power and undermined the effect size of our results, given that the incidence of cardiovascular disease was lower than among the participants that did not answer the questionnaire.

### 4.3. Implications

Public health practitioners, policymakers, and healthcare professionals should be aware that initiatives to support patients with low health literacy could be effective not only among patients with end-stage renal disease, but also in patients with earlier stages of CKD. Improving support to these patients, in particular with health-literacy-friendly strategies to improve the control of diabetes and obesity, could decrease the incidence of cardiovascular disease among CKD patients. Additionally, resources should be allocated to create programs and infrastructures that facilitate healthier lifestyles. This focus on lifestyle improvement, also known as lifestyle medicine, is known not only for facilitating disease treatment, but also for preventing the onset and progression of non-communicable chronic diseases, increasing quality of life, and reducing mortality and costs [34]. This is especially important when considering the tendency of the global population to live longer and cumulate disease-related complications along the life course.

Once lifestyle medicine is better integrated into routine clinical care, additional research will be needed to better evaluate the impact of lifestyle changes on CKD outcomes. Moreover, future studies should also take communicative and critical health literacy into consideration, as these have already been related to self-management skills in CKD [35].

## 5. Conclusions

Our study provides evidence of an association between low health literacy and the development of cardiovascular disease and shows that this association is mediated by uncontrolled diabetes and obesity. Hence, interventions to improve the control of diabetes and obesity, mainly by means of promoting a healthier lifestyle, could decrease the negative effects of low health literacy on CKD.

## Figures and Tables

**Table 1:** Comparison of baseline characteristics between participants with low and adequate health literacy in the CKD population.

Variables		Adequate Health Literacy (*n* = 1816)	Low Health Literacy (*n* = 926)	*p* Value
Demographic characteristics				
Sex % *of females*		54.6	57.9	0.104
Age *mean, (SD)*		52.7 (15.7)	58.0 (15.4)	<0.001
Education *%*	Low	3.2	11.8	<0.001
	Intermediate	63.6	77.4	
	High	33.1	10.9	
Monthly household income *%*	EUR < 1000	5.8	6.8	<0.001
	EUR 1000–3000	54.1	60.2	
	EUR > 3000	26.5	15.2	
	Information not given	13.7	17.8	
Clinical characteristics				
Cardiovascular disease *%*		9.6	17.0	<0.001
Mortality *%*		3.2	5.1	0.019
Uncontrolled hypertension *%*		50.0	54.2	0.037
Systolic blood pressure ^a^ *mean, (SD)*		133.0 (18.3)	136.0 (18.9)	<0.001
Diastolic blood pressure ^a^ *mean, (SD)*		76.5 (10.4)	76.5 (10.1)	0.890
Uncontrolled diabetes *%*		7.2	13.1	<0.001
HbA1c ^b^ *median (IQR)*		5.7 (0.5)	5.8 (0.6)	<0.001
Obesity *%*		23.0	27.6	0.007
BMI *mean, (SD)*		27.1 (4.8)	28.0 (4.8)	<0.001
Dyslipidemia *%*		21.8	24.7	0.078
Total cholesterol/HDL ratio *median (IQR)*		3.6 (1.7)	3.8 (1.8)	0.001
LDL ^c^ *mean, (SD)*		3.2 (1.0)	3.3 (1.0)	0.045
Albuminuria *%*		83.2	79.8	0.062
Albumin 24 h urine ^d^ *median (IQR)*		43.5 (47.8)	43.8 (49.9)	0.969
Current smoking *%*		18.2	19.8	0.303
CKD stage *%*	1	38.8	31.4	<0.001
	2	22.9	22.9	
	3	37.4	44.4	
	4	0.7	1.0	
	5	0.2	0.3	

CKD: Chronic kidney disease; BMI: body-mass index; HDL: high-density lipoprotein; LDL: low-density lipoprotein; ^a^ Blood pressure expressed in mmHg; ^b^ HbA1c expressed in percentage; ^c^ LDL expressed in mmol/L; ^d^ Albumin 24 h expressed in mg/24 h.

**Table 2:** Association between low health literacy and the CKD complications.

	Cardiovascular Disease			Mortality		
	OR	95% CI	*p* Value	OR	95% CI	*p* Value
Crude model	1.93	1.46–2.55	<0.001	1.59	1.08–2.36	0.020
Model adjusted for age and sex	1.76	1.31–2.23	<0.001	1.30	0.87–1.95	0.196

CKD: Chronic kidney disease. Values for health literacy, considering adequate health literacy as the reference.

**Table 3:** Percentage of the association between low health literacy, cardiovascular disease, and mortality explained by individual mediators.

		Percentage of the Association Explained			
Mediator	Model	Cardiovascular Disease	*p* Value	Mortality	*p* Value
Uncontrolled hypertension	Crude	0.6	0.649	3.4	0.310
	Adjusted ^a^	0.6	0.761	1.0	0.921
Uncontrolled diabetes	Crude	36.8	<0.001	44.7	0.001
	Adjusted ^a^	27.1	0.002	52.4	0.150
Obesity	Crude	8.0	0.027	4.8	0.274
	Adjusted ^a^	8.0	0.054	8.6	0.317
Dyslipidemia	Crude	2.3	0.247	1.9	0.541
	Adjusted ^a^	3.0	0.218	5.7	0.409
Albuminuria	Crude	9.9	0.086	16.3	0.090
	Adjusted ^a^	1.7	0.493	5.7	0.460

^a^ Model adjusted for age and sex.

## Data Availability

The datasets analyzed during the current study are available in the Lifelines Biobank repository, at https://www.lifelines.nl/researcher.

## Supplementary Materials

The following is available online at https://www.mdpi.com/article/10.3390/ijerph18105235/s1, Table S1: List of the medications used in the definition of treatment status for hypertension, diabetes, and dyslipidemia.
