# Supplementary material for: Do Uncontrolled Hypertension, Diabetes, Dyslipidemia, and Obesity Mediate the Relationship Between Health Literacy and Chronic Kidney Disease Complications?
Source: Int J Environ Res Public Health. 2021 May 14;18(10):5235. doi: 10.3390/ijerph18105235 (PMC8157126; doi:10.3390/ijerph18105235)
Supplement: Supplementary file 1 [file ijerph-18-05235-s001.zip › ijerph-1167764-supplementary.pdf]

Supplementary Material

Table S1: List of the medications used in the definition of treatment status for hypertension, diabetes, and dyslipidemia

Hypertension	Diabetes	Dyslipidemia
Diuretics= ATC-code C03A, C03B, C03C, C03E, C03X	Oral anti-diabetics= ATC-code A10B	Statins and fibrates= ATC-code C10A
Aldosterone antagonists= ATC-code C03D	Insulin= ATC-code A10A	
Beta-blockers= ATC-code C07		
Calcium antagonists= ATC-code C08		
ACE-inhibitors and angiotensin II antagonists = ATC-code C09		

ATC-code: Anatomical Therapeutic Chemical code
